# Optimizing maternal fat suppression with constrained image‐based shimming in fetal MR

**DOI:** 10.1002/mrm.27375

**Published:** 2018-07-29

**Authors:** Andreia S. Gaspar, Rita G. Nunes, Giulio Ferrazzi, Emer J. Hughes, Jana Hutter, Shaihan J. Malik, Laura McCabe, Kelly P. Baruteau, Mary A. Rutherford, Joseph V. Hajnal, Anthony N. Price

**Affiliations:** ^1^ Centre for the Developing Brain, School of Biomedical Engineering & Imaging Sciences King's College London, St Thomas' Hospital London United Kingdom; ^2^ Institute for Systems and Robotics/Department of Bioengineering, Instituto Superior Técnico Universidade de Lisboa Lisbon Portugal; ^3^ Instituto de Biofísica e Engenharia Biomédica Faculdade de Ciências da Universidade de Lisboa Campo Grande Lisbon Portugal; ^4^ Lysholm Department of Neuroradiology, National Hospital for Neurology and Neurosurgery University College London Hospitals NHS Foundation Trust London United Kingdom

**Keywords:** EPI, fat suppression, fetal imaging, image‐based shimming, SPIR

## Abstract

**Purpose:**

Echo planar imaging (EPI) is the primary sequence for functional and diffusion MRI. In fetal applications, the large field of view needed to encode the maternal abdomen leads to prolonged EPI readouts, which may be further extended due to safety considerations that limit gradient performance. The resulting images become very sensitive to water‐fat shift and susceptibility artefacts. The purpose of this study was to reduce artefacts and increase stability of EPI in fetal brain imaging, balancing local field homogeneity across the fetal brain with longer range variations to ensure compatibility with fat suppression of the maternal abdomen.

**Methods:**

Spectral Pre‐saturation with Inversion‐Recovery (SPIR) fat suppression was optimized by investigating SPIR pulse frequency offsets. Subsequently, fetal brain EPI data were acquired using image‐based (IB) shimming on 6 pregnant women by (1) minimizing B_0_ field variations within the fetal brain (localized IB shimming) and (2) with added constraint to limit B_0_ variation in maternal fat (fat constrained IB shimming).

**Results:**

The optimal offset for the SPIR pulse at 3 Tesla was 550 Hz. Both shimming approaches had similar performances in terms of B_0_ homogeneity within the brain, but constrained IB shimming enabled higher fat suppression efficiency.

**Conclusion:**

Optimized SPIR in combination with constrained IB shimming can improve maternal fat suppression while minimizing EPI distortions in the fetal brain.

## 1 INTRODUCTION

Functional and diffusion MRI of the fetus provide a means to study emerging human brain connectivity during its development in utero. Typically, echo planar imaging (EPI) is the preferred acquisition method because of its high temporal resolution.^1^, ^2^, ^3^ However, there are specific safety concerns when running EPI in the fetus, such as acoustic noise, peripheral nerve stimulation,^4^, ^5^ and specific absorption rate.^6^ Part of these restrictions can be accommodated by lowering the bandwidth in the phase encoding direction at the expense of increased sensitivity to susceptibility‐induced artefacts. This problem is further increased by the necessity to use single‐shot readouts to freeze fetal motion and the enlarged encoding matrix required to also cover the surrounding maternal tissue.

**Figure 1:**
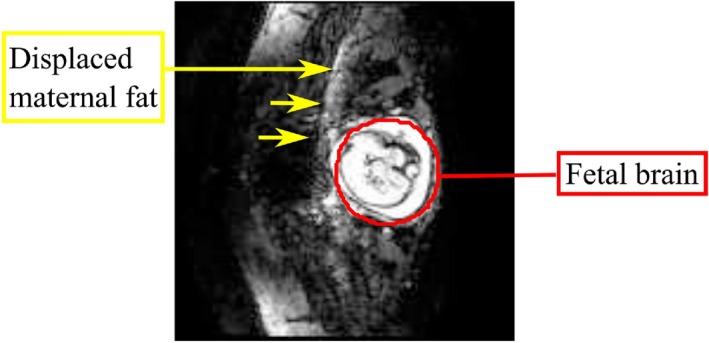
Example EPI image of the fetal brain (indicated in red) in which the spectral fat suppression was not able to eliminate fat signal from being superimposed on the fetal brain due to water fat shift

The bandwidth of the imaging sequence is inversely proportional to the water‐fat shift (WFS); high WFS in the context of fetal EPI is problematic, given that the subcutaneous maternal fat often appears superimposed on the fetal brain hindering functional and diffusion analyses (Figure 1). For this reason, effective suppression of the maternal fat signal is important.

Outer‐volume suppression is a possible solution, which requires the positioning of 2 or more saturation slabs.^7^ The posterior slab is easily positioned, but an anterior region is not straightforward because of the variation in maternal anatomy.

Alternatively, inner‐volume selection^8^, ^9^, ^10^ could be applied, but often the excitation requires composite pulses, not easily compatible with multiband, and slice acceleration, which is increasingly desired for functional imaging. The alternative is spectrally selective fat suppression techniques applied before excitation; these are valuable especially at high magnetic fields, where the difference between fat and water resonance frequencies is larger. One example is Spectral Pre‐saturation with Inversion Recovery (SPIR), which selectively saturates fat signals using a flip angle (FA) slightly above 90 º and a short recovery period so that the longitudinal magnetization of the fat is negligible at the start of the imaging sequence.^11^ With this method, the time needed before initiating the sequence is short, which is desirable in functional imaging. Nevertheless, its application requires a careful optimization of the parameters, which may not be adequate in all circumstances.^12^ In addition, it is important to ensure a reasonably homogeneous main magnetic field (B_0_) over the fat region to achieve effective saturation, but in the case of a highly localized B_0_ shim of the fetal brain, field homogeneity in the surrounding maternal tissues may be compromised.

Precise shimming in EPI acquisitions is important to avoid signal loss and minimize spatial distortions of the object in the image.^13^ Image‐based (IB) shimming techniques have been proved to be accurate and robust.^14^, ^15^ In localized IB shimming, an anatomically defined region of interest (ROI) is used to further improve homogeneity over that selected region.^16^ The optimization can be approached as a least squares problem in which the residual variations within that ROI are minimized.

To maximize performance, the shimmed region should be anatomically limited to the fetal brain. However, if we ignore distant areas such as maternal fat, their B_0_ variations will likely be increased, which, in turn, can compromise fat suppression. The use of 2 different shim settings: one optimized for fat suppression and the other applied during the imaging process is one possible strategy. However, this adds complexity and carries its own challenges because shim coils are generally not shielded and could generate eddy currents with long time constants when rapidly switched.

Some approaches have been previously presented to control B_0_ field outside the shimming ROI in the prostate, liver, and head.^15^, ^17^ The first consisted in weighting also a region of less interest in order to reduce its B_0_ dispersion and avoid possible shim degeneracy when the ROI was very small.^15^ The second method was implemented to optimize the B_0_ within the ROI while minimizing the number of pixels with deviation above a specific threshold (125 Hz at 3Tesla [T]) in the whole field of view (FOV).^17^, ^18^ This technique was later developed to include only the fat region and was applied in the liver.^19^ These approaches are variants of constrained IB shimming.

In this work, we focus on the use of a single static shim setting common for both fat suppression and EPI readout to overcome both susceptibility and WFS artefacts, and seek an optimal trade‐off between homogeneities of B_0_ over the fetal brain and maternal fat regions; the IB shimming method has the flexibility needed for this. We explore the feasibility of constrained IB shimming in combination with an optimized SPIR pulse for fetal imaging with EPI. In order to achieve robust shimming, an IB shimming tool was developed to minimize magnetic field inhomogeneity, and 2 approaches were compared: localized IB (L‐IB) shimming in which B_0_ was optimized for homogeneity in the fetal brain only; and fat constrained IB (FC‐IB) shimming in which optimization of the brain region was combined with constraints across maternal fat regions.

## 2 METHODS

### 2.1 Data acquisition

MR acquisition was performed using a 3T Philips Achieva scanner with a 32‐channel cardiac receive coil wrapped around the maternal abdomen. For methods development, 7 fetuses ranging from 27 to 31 weeks in gestation (maternal body mass index range from 21.7 to 30.6) were scanned. Two fetuses were scanned to optimize the SPIR pulse, and 6 were scanned with both localized and fat constrained IB shimming.

B_0_ field maps were acquired using a rapid spoiled gradient echo sequence with 2 interleaved echoes (TE1/TE2 = 4.6/6.9 ms) chosen so that the water and fat signals were in phase. B_0_ field maps with zero shims were acquired in all examinations and used as the input data for the IB shimming calculations. Additional fat‐water images using 3‐point Dixon (TE1/TE2/TE3 = 4.6/5.6/6.6 ms) were acquired in order to aid segmentation of the fat regions.^20^ Acquisition times for B_0_ field map and Dixon were 14 and 20 seconds, respectively. B_0_ and Dixon acquisitions, with matched geometry to facilitate fat segmentation, were performed with multiple sequential 2D scanning (FOV of 350 × 350 × 100 mm^3^; acquired resolution of 5 × 5 × 10 mm^3^ and reconstructed resolution of 2.5 × 2.5 × 10 mm^3^).

All the optimization was performed using Matlab (The MathWorks, Inc., Natick, MA) on a separate computer next to the main console. Once calculated, shim values were automatically propagated to all following scans. EPI images were acquired at 2.2‐mm isotropic resolution for a FOV of 320 × 320 mm^2^, with in‐plane SENSitivity Encoding acceleration (R = 2), transverse orientation to the scanner. Gradient performance was limited to reduce acoustic noise predominantly by using a fixed readout switching frequency of 490 Hz, previously determined as a good operating point on the system;^21^ this resulted in an WFS of 33 pixels in the phase direction.

Finally, all scan acquisitions were in compliance with ethical requirements, and written informed consent was obtained from all the participants.

### 2.2 SPIR frequency offset

The default vendor SPIR pulse was chosen because of its good compromise between pulse length, frequency profile, and specific absorption rate. Default parameters used for the SPIR pulse were: bandwidth of 752 Hz, duration 7.5 ms, frequency offset 635 Hz, and time‐bandwidth product (TBP) of 6.20. The FA of approximately 110 º was adjusted for the inversion time and repetition time per shot.

In order to investigate optimal settings for the SPIR pulse, its spectral profile was simulated and compared with the frequency histograms of fat and water regions after shimming to predict whether fat suppression should be successful.

Effectiveness of fat suppression was evaluated in vivo by systematically changing the frequency offset of the SPIR pulse in 2 subjects. The center frequency of the pulse was shifted in steps of 40 Hz, between 743 and 483 Hz so moving the suppression band toward the water resonance. The optimized value was chosen as the best compromise between effective suppression of fat regions and retention of water signal from the fetal brain. This optimized value was compared against the default scanner value for all the following shim tests.

### 2.3 IB shimming algorithm

The IB shimming algorithm was developed in Matlab (2012b; The MathWorks, Inc.) and included ROI definition, phase unwrapping, and shim calculation.

The definition of an ROI consisted in circumscribing the fetal brain within an ellipsoid of adjustable position and size. The ROI was semiautomatically set in 1 slice and then propagated to all the other slices. Phase unwrapping based on the Goldstein 2D method could be performed when required by the user.^22^

Localized shimming consisted in a least square solution, which minimizes the B_0_ inhomogeneity ( $\Delta B_{0}$) within the brain region regularized by the shim parameters (*s*) (Equation 1). The small dimension of the fetal head could increase the probability of ill‐conditioned optimization and thus to unstable solutions in a nonconstrained IB shimming algorithm. In order to accommodate this problem, a Tikhonov regularization term (with λ=0.03) and upper and lower constraint on the solution ( lb≤s≤ub) were included for the localized IB shimming.^23^

The constrained IB shimming minimizes the same cost function as localized IB shimming—this time adding linear constraints to the fat regions, which were segmented by thresholding the fat image obtained with the Dixon acquisition. This constraint can be defined as:

(1) $$\begin{array}{c} s=min_{s}{\left\|A\left(r_{brain}\right)s-\Delta B_{0}\left(r_{brain}\right)\right\|}_{2}^{2}+\lambda {\left\|s\right\|}_{2}^{2}\\ s.t.\{\begin{array}{c} -Cs\leq d_{1}\\ Cs\leq d_{1}\\ lb\leq s\leq lu \end{array} \end{array}$$

where $A\epsilon {\mathbb{R}}^{N_{b}\times N_{s}}$ is a matrix that encodes the spatial distribution of the spherical harmonics of first and second order for the shim coils, $N_{b}$ is the number of pixels in the ROI, and $N_{s}$ is the number of spherical harmonics. The variable $r_{brain}\epsilon {\mathbb{R}}^{N_{b}}$ corresponds to the spatial coordinate of each pixel and ranges over the ROI. The matrix $C\epsilon {\mathbb{R}}^{N_{f}\times N_{s}}$ encodes the spherical harmonic distribution of the shim coils in the fat regions ( $N_{f}$ is the number of pixels in the fat region). The vectors $d_{1}$ and $d_{2}$ represent the frequency constraints for each pixel in the fat region, ( $d_{1}$ = –350 Hz; $d_{2}$ = 100 Hz) and were defined from simulation as the effective range for the SPIR pulse used at the optimum determined frequency offset (note: the absolute range of fat being offset by −430 Hz). These limits were chosen considering the 95% peak limits for the pulse envelope obtained from Bloch simulation of the optimized SPIR pulse.

Calculation of the shim currents up to second order was performed using the function *lsqlin* from Matlab (The MathWorks, Inc.) 24 and were constrained to the lower ( lb) and upper ( ub) bound limits allowed by the hardware. The residual B_0_ field map was always simulated by projecting shim currents onto the field maps acquired with zero shim and measured in order to verify effectiveness.

## 3 RESULTS

### 3.1 Fat suppression

**Figure 2:**
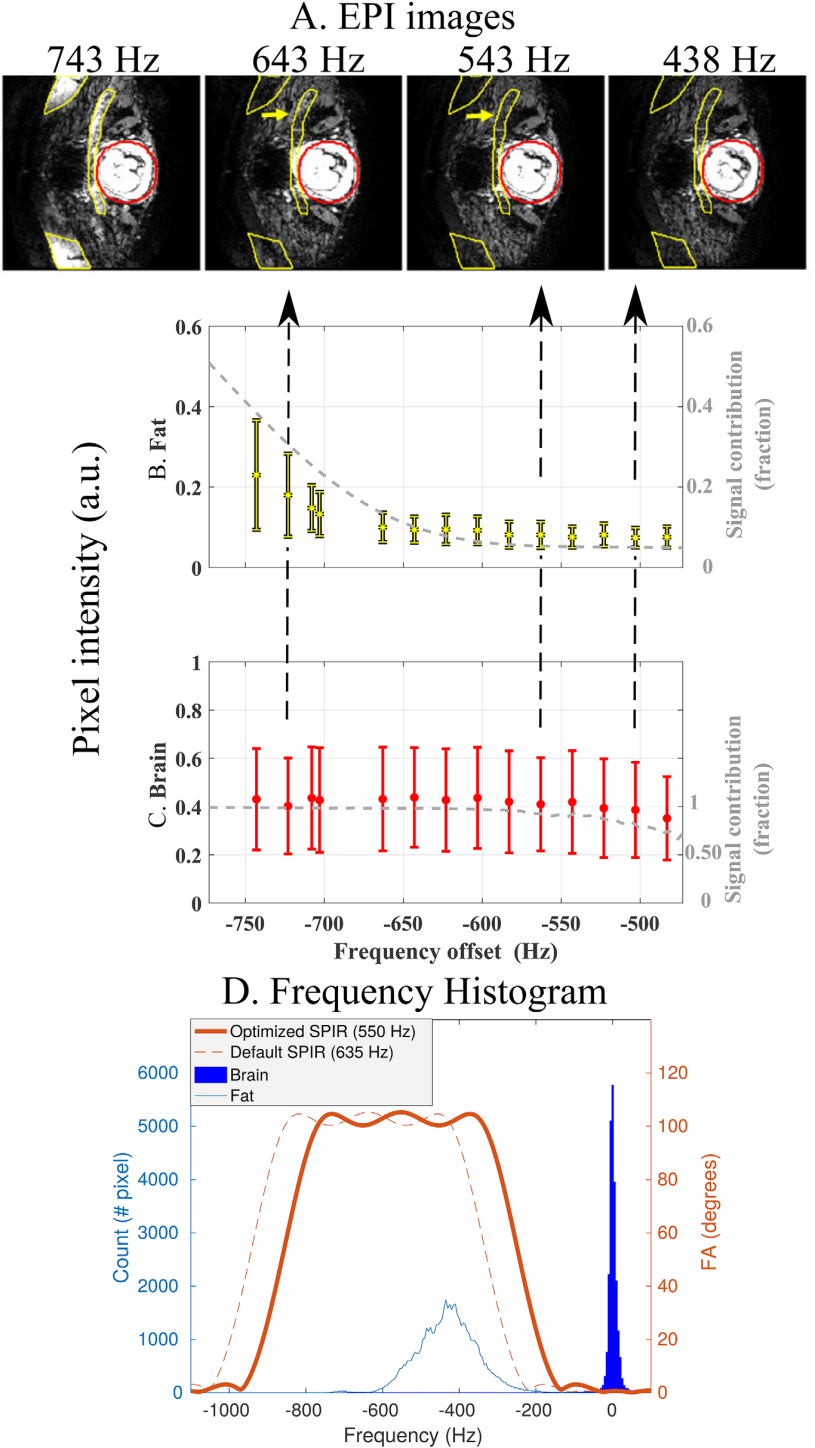
Effect of SPIR frequency offset on fat and water signal with localized IB shimming, of a representative subject. EPI images in (a) and graphs of the mean pixel intensity in (b) areas of fat artefact (yellow) and (c) fetal brain regions (red) for different frequency offsets. Fat ROI was delineated based on the EPI image with SPIR offset of 743 Hz, and brain ROI was the same region used in IB shimming. With an offset of 643 Hz, it is possible to observe residual fat, which is suppressed with an offset of 543 Hz. A further decrease of the SPIR offset to 483 Hz is associated with decrease of the fetal brain signal. The gray line in each panel represents, respectively, the simulated signal of fat (b) and water (c) as a function of the SPIR pulse offset. The variation of the simulated signal is in good agreement with the measurement results. In (d), fat (light blue) and water (dark blue) frequency histograms are superimposed with simulated SPIR pulse with optimized (550 Hz, thick orange line) and default (635 Hz, dashed orange line) offset frequency settings. a.u., arbitrary units

The increment in the SPIR frequency offset did not have a significant impact on the mean water signal within the fetal brain up to ∼550 Hz, whereas the fat signal reached its minimum at that frequency (Figure 2). This is confirmed in the simulation presented in Figure 2d, in which a frequency offset of 550 Hz suggests improved fat suppression, compared to 635 Hz, but still without water saturation. Figure 2c shows that a further decrease to 483 Hz (right) results in lower signal in specific brain regions, which is corroborated by simulations of the signal intensity for different SPIR pulse offsets. Corresponding EPI images in the fetal brain, as shown in Figure 2a, demonstrate the improvement with varying SPIR pulse settings on maternal fat suppression.

### 3.2 IB shimming algorithm

The standard deviation (SD) of the measured frequencies within the fetal brain ROI (n = 6) with zero shim was 36 ± 12 Hz. The reduction of field inhomogeneity when applying localized (SD = 10.9 ± 5.7 Hz) or constrained (SD = 11.7 ± 5.9 Hz) shimming was very similar. When applying the fat constrained IB shimming, the percentage of the fat region outside the linear frequency constraints decreased to 8 ± 4% when compared with 15 ± 11% with localized IB shimming.

**Figure 3:**
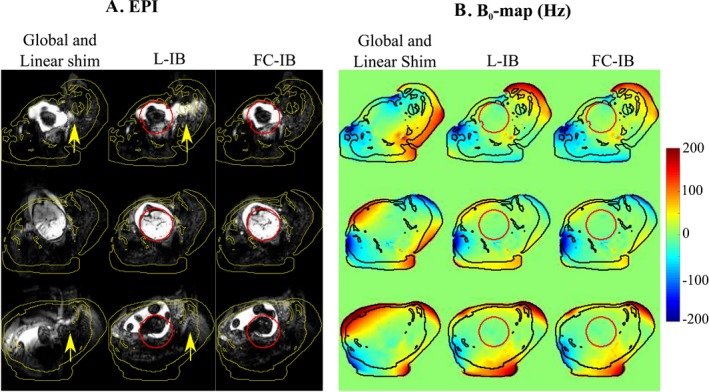
EPI and residual B_0_ field frequency of the fetal brain in 3 slices of the same subject. (a) EPI in the fetal brain using (from left to right): global (full FOV) shim using only linear terms, localized IB shimming (L‐IB), and fat constrained IB shimming (FC‐IB) with optimal SPIR. (b) Residual B_0_ field frequency at the same position for global linear shim, L‐IB shimming, and FC‐IB shimming. The yellow arrows outline residual fat. Yellow lines delimit the regions determined by thresholding the Dixon images, which also represent fat in undistorted space. In the B_0_ field maps, the fat is outlined in black and the ROI of the fetal brain is shown in red

Figure 3 shows how the inclusion of localized and high‐order shim is important to avoid local spatial distortions. B_0_ field maps for the same subject (gestational age of 37 + 2 weeks) show that it is possible to limit the frequency in fat regions without extensively compromising B_0_ homogeneity in the brain. In addition, combination of constrained IB shimming with optimized SPIR yields effective removal of fat artefacts.

**Figure 4:**
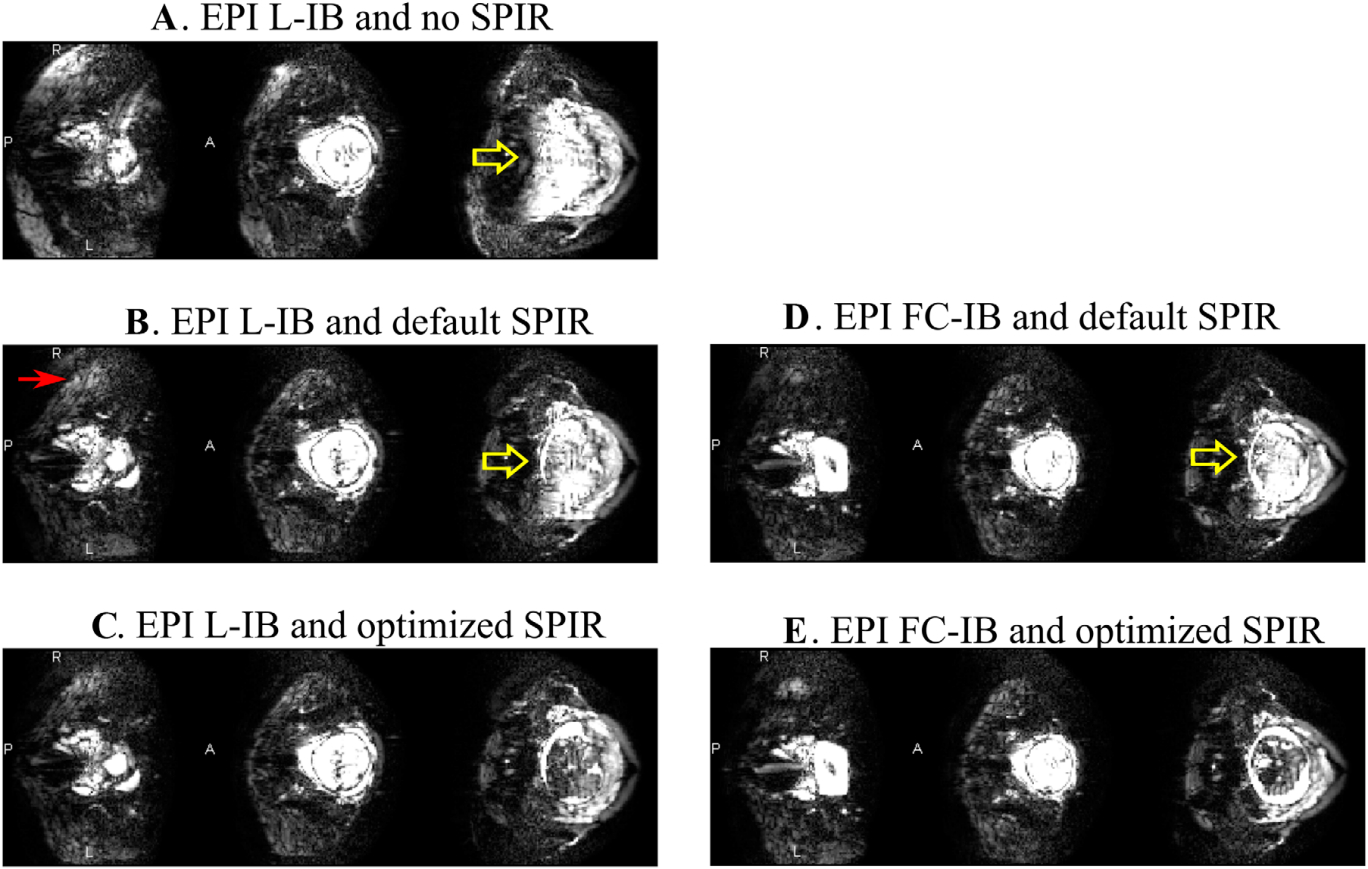
Fetal EPI images (scaled‐up intensity window) in the same volunteer with different combinations of IB shimming and SPIR pulse offsets. The yellow arrow points to the residual fat superimposed onto the fetal brain, and the red arrow indicated unsuppressed fat in peripheral regions. The first column (a,b,c) corresponds to localized image‐based shimming (L‐IB). The second column (d,e) corresponds to the fat constrained IB shimming (FC‐IB). The use of the optimized SPIR (c,e) is associated with a visible reduction of fat artefact. The additional association of the optimized frequency offset (550 Hz) with the constrained IB shimming (e) further reduces the presence of fat showing the robustness of the method

Figure 4 shows that EPI data quality improves in terms of fat suppression when applying the optimized SPIR pulse, with a further improvement when used in combination with constrained IB shimming.

**Figure 5:**
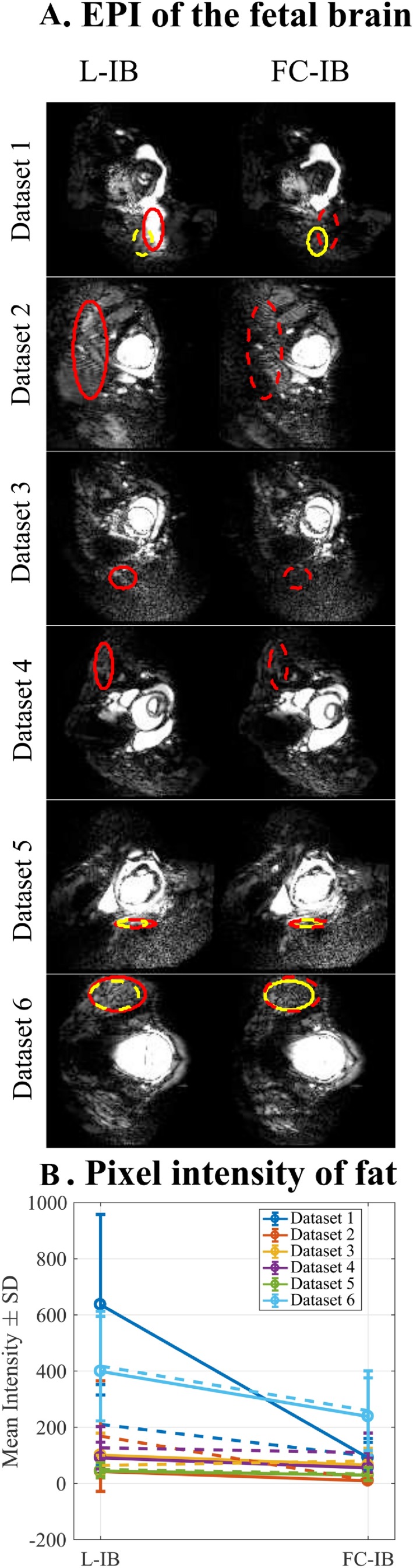
Examples of residual fat artefact in (a) EPI of the fetal brain after applying localized (L‐IB) and fat constrained (FC‐IB) image‐based shimming, across 6 subjects. ROIs were delineated in the worst slice in terms of residual fat for both L‐IB (red) and FC‐IB data sets (yellow) independently, resulting in 12 selected ROIs (only slices containing L‐IB selected regions are shown here). The mean and SD of the signal in L‐IB (solid line) and FC‐IB (dashed line) selected regions are shown in (b)

Figure 5 shows EPI fetal imaging acquired with L‐IB and FC‐IB shimming with optimized SPIR for 6 subjects (see also Supporting Information Figure S1). Within the subject group tested, there is no evidence of a detrimental effect on spatial distortion when applying FC‐IB shimming; data sets 1, 2, and 6 show a trend for lower signal intensity of fat with FC‐IB shimming, whereas 3, 4, and 5 did not show measurable differences in signal intensity between methods.

## 4 DISCUSSION

Performing optimal B_0_ shimming is an important step when using low‐bandwidth acquisitions such as EPI. This is even more critical in fetal applications, where the enhanced safety constraints and large FOVs require long readouts. In this work, two methods for IB shimming—localized (L‐IB) and fat constrained (FC‐IB)—were implemented and studied to minimize these problems; both allowed for locally optimized shimming of the fetal brain, but the second method included an additional constraint on the B_0_ variation in surrounding maternal tissue to improve effectiveness of fat suppression.

The interaction between localized IB shimming and default SPIR settings often leads to incomplete fat suppression. Therefore, the compatibility of these two techniques was improved by two means: initially, the SPIR pulse was optimized to effectively saturate the fat signal while not damaging the water signal. Subsequently, the optimized SPIR pulse was paired with the constrained IB shimming, which provides good shim performance over the fetal brain while keeping the maternal fat signal under controlled conditions.

The frequency offset that showed the best trade‐off between fat suppression and negligible water signal attenuation, under localized shimmed conditions, was 550 Hz, which is similar to the one presented in a previous study.^12^ In addition, our findings showed that an offset of 550 Hz is a conservative threshold because water signal was affected only when using lower offsets. In fact, the narrow linewidth of water within the fetal brain region allows having lower SPIR pulse offsets in comparison to other applications.

The presented dispersion metrics showed that the improvement in terms of B_0_ field homogeneity within the fetal brain ROI was very similar between L‐IB and FC‐IB shimming, demonstrating that there was minimal penalty in order to achieve effective fat suppression. Although the shimming result is not significantly affected by respiratory motion (Supporting Information Figure S2 and Supporting Information Video S1) or an active fetus (Supporting Information Figure S3 and Supporting Information Video S2), the achievable B_0_ field homogeneity for both L‐IB and FC‐IB shimming was limited in 2 cases by the existence of intestinal gas near the vicinity of the fetal brain. This leads to high field variations not possible to correct for with conventional shim hardware.

The IB shimming method can be integrated into a fetal exam with only a small increase in protocol time. The required B_0_ field map takes 14 seconds; the brain ROI delineation and phase unwrapping usually take 1 minute, which can be performed while less‐shim dependent sequences are acquired. The additional time required for FC‐IB shimming results from the Dixon acquisition and fat segmentation and is approximately 20 seconds.

Although in this study increased fat suppression and shim performances are demonstrated when compared with the default settings, this work is open to further improvements. One limitation relates to the choice of SPIR pulse shape used and the trade‐off between a well‐defined frequency profile and limiting the length of the pulse. It could be beneficial to use a pulse with a higher time‐bandwidth product, which could provide a flatter band‐pass profile and a sharper transition without side bands to allow an even closer frequency offset to be utilized.

Furthermore, unlike methods using adiabatic pulses, the SPIR pulse is sensitive to variations in B_1_, which can vary substantially when imaging the fetus at 3T (Supporting Information Figure S4). This can lead to variations in FA and ultimately to incomplete fat suppression, which may be responsible for some residual fat signal even when all the fat frequencies are within the SPIR pulse bandwidth.^25^ One option would be to apply the SPectral Attenuated Inversion Recovery method,^26^ which uses adiabatic inversion and is less sensitive to the B_1_ inhomogeneities, but it requires long inversion times leading to an undesirable increase in acquisition dead time. Alternatively, improvements could be gained by also performing B_1_ mapping and calculating dedicated radiofrequency (RF) shim settings for the SPIR pulse, although these are likely to be quite different to those optimal for the fetal brain region alone, and thus the ability to switch to different RF shims for the imaging RF pulses would likely be required.

## 5 CONCLUSION

The presented study demonstrates the importance of a robust approach to perform shimming in fetal EPI applications to minimize susceptibility and WFS artefacts. This work shows how the synergetic combination of IB shimming methods with an optimized SPIR pulse for fat suppression can achieve this goal.

## Supporting information

Additional supporting information can be found in the online version of this article.

**FIGURE S1**. One example of good fat suppression where EPI of the fetal brain acquired with L‐IB and optimal SPIR offset is presented in different orientations (L, left; R, right; A, anterior; P, posterior; I, inferior; S, superior). In this example, only water in maternal tissue is contributing to the signal
**FIGURE S2**. Effect of maternal respiration in the ΔB_0_ field map within the fetal brain: (a) 1 frame of a dynamic B_0_ field map with delineated fetal brain ROI (orange), and fat regions (green) and surrogate marker of respiratory motion in the diaphragm (blue); (b) evolution of the mean ΔB_0_ field map ± SD (Hz) within the brain (orange), and fat (green) alongside the line profile indicating diaphragm movements. Respiratory motion can be observed in Supporting Information Video S1
**FIGURE S3**. Effect of fetal motion on the ΔB_0_ field map within the fetal brain: (a) 1 frame of a dynamic B_0_ field map with delineated fetal brain ROI (orange), and fat region (green); (b) evolution of the mean ΔB_0_ field map ± SD (Hz) within the brain ROI and fat (green) along acquisition time. Fetal head motion can be observed in Supporting Information Video S2
**FIGURE S4**. B_1_ map of the fetus (% of nominal FA achieved), also showing maternal fat region delineated in black

**VIDEO S1**. Dynamic B_0_ field map showing the effect of respiratory motion on B_0_ field homogeneity. Thirty frames with 1.27 s/frame are presented. The magnitude image is presented on the left, and the brain ROI is delineated in orange

**VIDEO S2**. Dynamic B_0_ field map showing the effect of fetus motion on B_0_ field homogeneity. Thirty frames with 1.27 s/frame are presented. The magnitude image is presented on the left, and the brain ROI is delineated in orange
