# Supplementary material for: Optimizing maternal fat suppression with constrained image‐based shimming in fetal MR
Source: Magn Reson Med. 2018 Jul 29;81(1):477–85. doi: 10.1002/mrm.27375 (PMC6282825; doi:10.1002/mrm.27375)
Supplement: Supplementary file 1 — FIGURE S1. One example of good fat suppression where EPI of the fetal brain acquired with L‐IB and optimal SPIR offset is presented in different orientations (L, left; R, right; A, anterior; P, posterior; I, inferior; S, superior). In this example, only water in maternal tissue is contributing to the signal FIGURE S2. Effect of maternal respiration in the ΔB0 field map within the fetal brain: (a) 1 frame of a dynamic B0 field map with delineated fetal brain ROI (orange), and fat regions (green) and surrogate marker of respiratory motion in the diaphragm (blue); (b) evolution of the mean ΔB0 field map ± SD (Hz) within the brain (orange), and fat (green) alongside the line profile indicating diaphragm movements. Respiratory motion can be observed in Supporting Information Video S1 FIGURE S3. Effect of fetal motion on the ΔB0 field map within the fetal brain: (a) 1 frame of a dynamic B0 field map with delineated fetal brain ROI (orange), and fat region (green); (b) evolution of the mean ΔB0 field map ± SD (Hz) within the brain ROI and fat (green) along acquisition time. Fetal head motion can be observed in Supporting Information Video S2 FIGURE S4. B1 map of the fetus (% of nominal FA achieved), also showing maternal fat region delineated in black [file MRM-81-477-s001.docx]

**Optimizing maternal fat suppression with constrained image-based shimming in fetal MR.**

Andreia S Gaspar ^1,2,3^, Rita G Nunes ^1,2,3^ , Giulio Ferrazzi ^1^ , Emer J Hughes ^1^ , Jana Hutter^1^, Shaihan J Malik ^1^ , Laura McCabe ^1^ , Kelly P Baruteau ^1,4^ , Mary A Rutherford ^1^ , Joseph V Hajnal^1^, and Anthony N Price^1^.

^1^ Centre for the Developing Brain, School of Biomedical Engineering & Imaging Sciences, King's College London, St Thomas' Hospital, Westminster Bridge Rd, London SE1 7EH, UK

^2^ Institute for Systems and Robotics / Department of Bioengineering, Instituto Superior Técnico, Universidade de Lisboa, Lisbon, Portugal

^3^ Instituto de Biofísica e Engenharia Biomédica, Faculdade de Ciências da Universidade de Lisboa, Campo Grande, 1749-016, Lisbon, Portugal

^4^Lysholm Department of Neuroradiology, National Hospital for Neurology and Neurosurgery, University College London Hospitals NHS Foundation Trust.

**Supporting Information**

- 1. **EPI image**

Fat suppression can be fully achieved with the optimized SPIR pulse offset in combination with image-base shimming. An example of good fat suppression is presented in Supporting Information Figure S1.


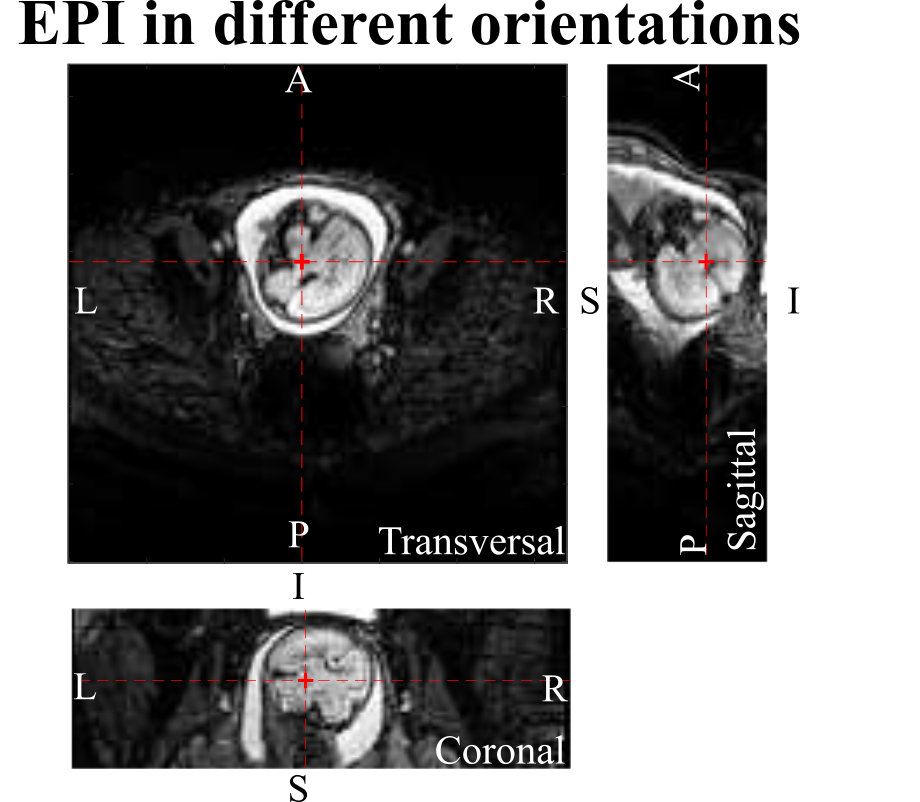


**Supporting Information Figure S1:** One example of good fat suppression where EPI of the fetal brain acquired with L-IB and optimal SPIR offset is presented in different orientations (L-Left; R-right; A-anterior; P-Posterior; I-Inferior; S-Superior). In this example, only water in maternal tissue is contributing to the signal.

- 1. **Effect of motion on B_0_ field homogeneity**

To evaluate the effect of motion on the methodology presented, we include two dynamic B_0_ field maps (30 frames – 1.27 s per frame). The first shows the effect of maternal respiration on the B_0_ field within the fetal brain ROI, and fat region (Supporting Information Figure S2 and Video S1). In Supporting Information Figure S2b) we can see that even with a large superior-inferior displacement close to the diaphragm, the mean ΔB_0_ in the fetal brain is maintained within ~5 Hz range. The variation of B_0_ field within the delineated fat region is also maintained within similar values along time.

The second example shows the effect of fetal motion on the mean ΔB_0_ within the brain ROI. This is shown in Supporting Information Figure S3 and Video S2. Even with sharp movement of the baby, the mean B_0_ is stable within 1-2 Hz.


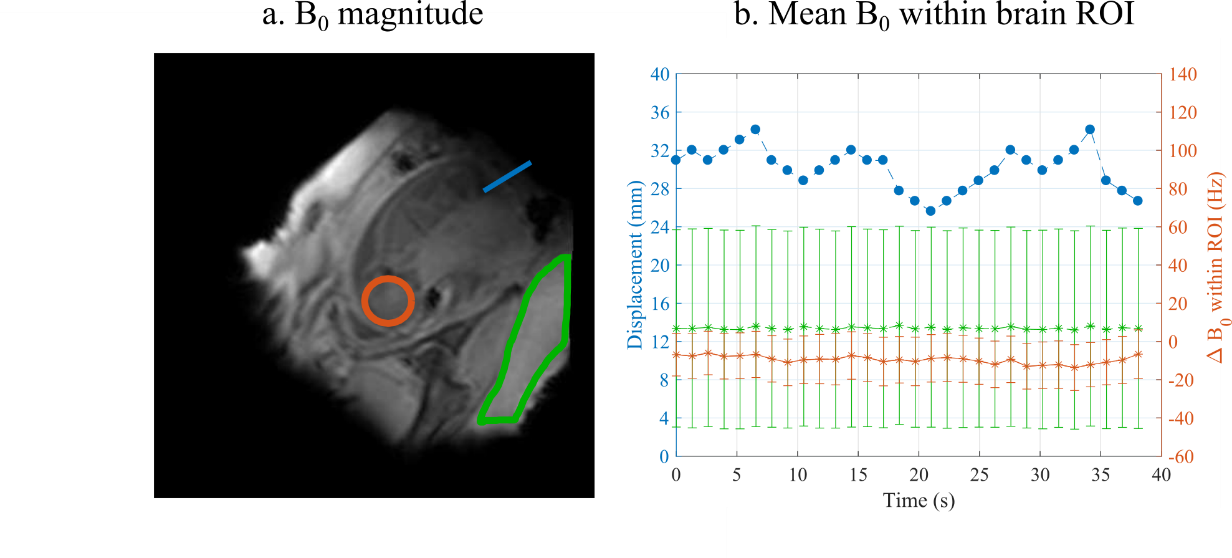


**Supporting Information Figure S2:** Effect of maternal respiration in the ΔB_0_ field map within the fetal brain: a) one frame of a dynamic B_0_ field map with delineated fetal brain ROI (orange), and fat regions (green) and surrogate marker of respiratory motion in the diaphragm (blue); b) evolution of the mean ΔB_0_ field map ± Standard Deviation (Hz) within the brain (orange), and fat (green) alongside the line profile indicating diaphragm movements. Respiratory motion can be observed in Supporting Information Video S1.


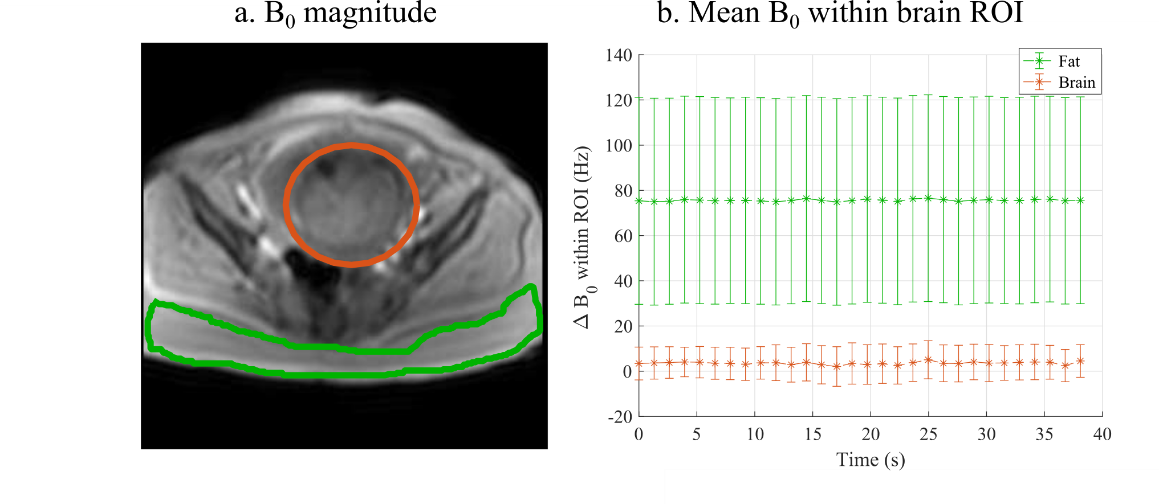


**Supporting Information Figure S3:** Effect of fetal motion on the ΔB_0_ field map within the fetal brain: a) one frame of a dynamic B_0_ field map with delineated fetal brain ROI (orange), and fat region (green); b) evolution of the mean ΔB_0_ field map ± Standard Deviation (Hz) within the brain ROI and fat (green) along acquisition time. Fetal head motion can be observed in Supporting Information Video S2.

- 1. **B_1_ field effect in fetal imaging**

The B_1_ field is also an important consideration in fetal imaging at 3T in order to evaluate the effectiveness of the SPIR pulse. In fact, the B1 field often exhibits high variation within the fetus due to the interactions of both the maternal tissue and surrounding amniotic fluid. However, B1 within maternal fat regions is often close to nominal B1, but some variations within this region are expected. A B1 map using the DREAM (1) method is presented in Supporting Information Figure S4 covering the fetal brain and surrounding maternal tissue to demonstrate typical field patterns.


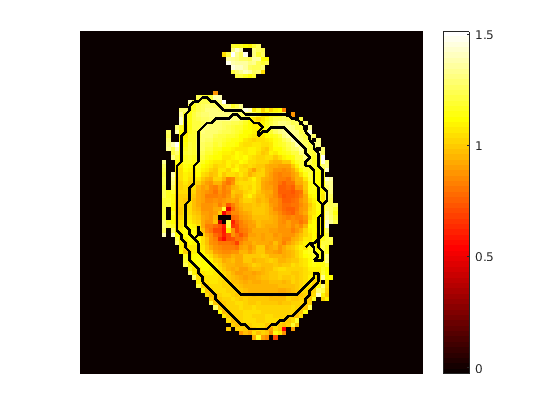


**Supporting Information Figure S4:** B_1_ map of the fetus (% of nominal flip angle achieved), also showing maternal fat region delineated in black.

**References – Supporting Information**

1. Nehrke K, B P. DREAM—A Novel Approach for Robust, Ultrafast, Multislice B1 Mapping. Magn Reson Med . 2012;68:1517–26.
